# Optimizing the Expression of Human Dopamine Receptors in *Escherichia coli*

**DOI:** 10.3390/ijms22168647

**Published:** 2021-08-11

**Authors:** Vanessa Boritzki, Harald Hübner, Anni Allikalt, Peter Gmeiner, Birgitta M. Wöhrl

**Affiliations:** 1 Department of Biochemistry IV—Biopolymers, Universität Bayreuth, Universitätsstr. 30, 95447 Bayreuth, Germany; vanessa.boritzki@gmx.net; 2 Department of Chemistry and Pharmacy, Medicinal Chemistry, Friedrich-Alexander-Universität Erlangen-Nürnberg, Nikolaus-Fiebiger Str. 10, 91058 Erlangen, Germany; harald.huebner@fau.de (H.H.); anni.allikalt@fau.de (A.A.); peter.gmeiner@fau.de (P.G.)

**Keywords:** human dopamine receptors, expression in *E. coli*, GPCR, protein engineering, FACS, radioligand binding, fluorescent ligand, gene library

## Abstract

The human dopamine receptors D_2S_ and D_3_ belong to the group of G protein-coupled receptors (GPCRs) and are important drug targets. Structural analyses and development of new receptor subtype specific drugs have been impeded by low expression yields or receptor instability. Fusing the T4 lysozyme into the intracellular loop 3 improves crystallization but complicates conformational studies. To circumvent these problems, we expressed the human D_2S_ and D_3_ receptors in *Escherichia coli* using different N- and C-terminal fusion proteins and thermostabilizing mutations. We optimized expression times and used radioligand binding assays with whole cells and membrane homogenates to evaluate K_D_-values and the number of receptors in the cell membrane. We show that the presence but not the type of a C-terminal fusion protein is important. Bacteria expressing receptors capable of ligand binding can be selected using FACS analysis and a fluorescently labeled ligand. Improved receptor variants can thus be generated using error-prone PCR. Subsequent analysis of clones showed the distribution of mutations over the whole gene. Repeated cycles of PCR and FACS can be applied for selecting highly expressing receptor variants with high affinity ligand binding, which in the future can be used for analytical studies.

## 1. Introduction

G protein-coupled receptors (GPCRs) are intensely studied drug targets since they regulate many physiological processes [1]. Approximately 35% of all approved drugs target GPCRs [2]. They are integral membrane proteins, harboring seven transmembrane helices that are connected by alternating intra- and extracellular loops with an extracellular N- and an intracellular C-terminus. GPCRs are highly flexible and can assume many different conformations [3]. They mediate cellular responses to hormones and neurotransmitters. Upon ligand binding to a GPCR on the cell surface, the receptor undergoes conformational changes thereby transmitting the signal from the extracellular space to intracellular proteins like G proteins or arrestins which in turn activate various signaling pathways in the cell [4].

The human dopamine receptors D_2_ and D_3_ also belong to the GPCRs [5]. Both dopamine receptors are located in the central nervous system and are stimulated by the endogenous ligand dopamine [6]. They are well-established therapeutic targets for serious neurological disorders. In order to understand the exact molecular mechanisms of signal transduction of GPCRs, it is necessary to elucidate the activation mechanism of the receptors. This would contribute significantly to the development of new drugs that bind with high specificity to the respective receptor subtypes, thereby reducing side effects to a minimum (subtype selectivity). This requires structural and biochemical investigations of the receptors in their inactive and active conformations as well as in potential transitional states. 

Although more than 800 different GPCRs are known, structures of only ~95 unique GPCRs are available so far and studies that require purified protein are quite rare (https://gpcrdb.org/structure/statistics (accessed on 21 May 2021)) [7,8]. The conformational flexibility of GPCRs has made it difficult to obtain crystal structures or to observe the actual conformational changes, i.e., by NMR spectroscopy, that are responsible for signal transduction. One of the greatest challenges is to produce recombinant, stable receptors in sufficient quantities for structural and biological analyses. Expression of GPCRs in eukaryotic systems, i.e., yeast, insect, or mammalian cells, is well established and suitable if investigations are performed that require a similar or even the identical environment in which GPCRs naturally occur. Eukaryotic systems are often used if the presence of a G protein or posttranslational modifications are necessary or if the GPCR is in need of the natural lipid composition for folding and function. However, expression of the receptors in *Escherichia coli* (*E. coli*) is easier to handle and can be used if posttranslational modifications are not desired. Furthermore, mutations can be introduced easily and expression levels can be optimized before purification of larger amounts of protein [9].The bacterial system also allows for specific isotope labeling of amino acids, which can then be used for the observation of conformational changes by NMR spectroscopy. On the contrary, specific amino acid labeling and growth in deuterated media is expensive in insect and mammalian cells and reduces their viability [10,11,12]. Independent of the expression system, working with the wild type receptor is almost impossible due to low expression and/or instability of the protein during and after purification. However, inserting mutations into the receptor gene can have a great impact on the functional incorporation of the receptor into the cell membrane, the overall expression level, and thermostability [13]. In general, the consequences of amino acid exchanges in proteins can be easily and quickly tested in *E. coli*. 

However, the heterologous expression and selection of GPCRs in *E. coli* has been shown for only a limited number of GPCRs and can encounter several obstacles. For example, the leukotriene B4 receptor BLT2 and the ghrelin receptor were overexpressed in *E. coli* in inclusion bodies and successfully refolded into their native state [14,15]. In contrast, the neurotensin receptor NTR1, the tachykinin receptor NK1, the α_1A_ and α_1B_ adrenergic receptors, the human adenosine receptor A_2A_, and the cannabinoid receptors CB_1_ and CB_2_ could be detected as correctly folded proteins in the *E. coli* inner membrane [16,17,18,19,20,21,22]. The NTR1 and the α_1B_ adrenergic receptors could even be purified successfully from *E. coli* in sufficient amounts to carry out structural experiments like crystallography or solution NMR [23,24].

Common to all receptors expressed in *E. coli* was that the expression constructs as well as the expression conditions had to be determined individually. Most likely, this is because of specific properties of the individual receptors which have not been properly identified so far. Moreover, purification of the receptors in the mg-range necessary for structural studies was only achieved after protein engineering and especially by insertion of stabilizing amino acid exchanges.

Until now, only the wild type gene of the human dopamine receptors D_2_ and D_3_ could be expressed in *E. coli*. However, their expression level was much too low to purify sufficient amounts of proteins, let alone to perform structural studies with them [25]. Obviously, based on previous experience with other receptors, new expression strategies should be developed and established for the two dopamine receptors in *E. coli* in order to optimize their expression levels and obtain reasonable amounts of functional and stable receptor.

In this study, we performed a thorough analysis of the expression conditions for the two human dopamine receptors D_2_ and D_3_ in *E. coli*. We optimized the constructs and growth conditions and were able to considerably improve the presence of correctly folded receptor molecules in the *E. coli* membrane tested by a radioligand binding assay. In addition, we established a mutagenesis and screening system to identify improved dopamine receptor variants by applying a fluorescence-based setup. Our protocols can be used to pursue various approaches, e.g., screening of new ligands, identification and characterization of new receptor variants that might be more stable, fluorescence-activated cell sorting (FACS) analysis with a fluorescent ligand or specific isotope labeling of the receptors for NMR studies to investigate conformational changes. 

## 2. Results and Discussion 

### 2.1. Expression of the Human D_2S_ Receptor in E. coli Using N- and C-Terminal Fusion Proteins

**Figure 1:**
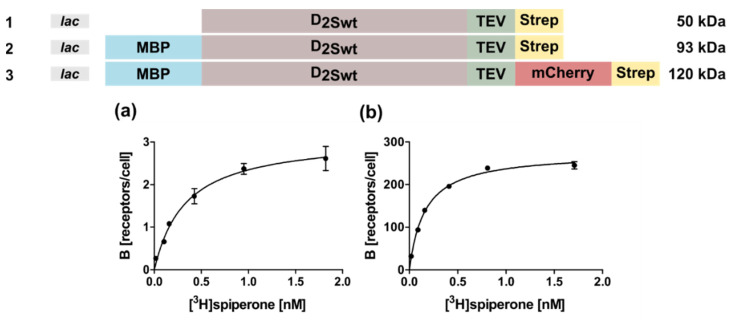
D_2S_ wild type constructs used for expression in *E. coli* and tested in radioligand binding assays with whole cells. The constructs tested in *E. coli* are shown on top. Only with constructs 2 and 3 binding of the ligand [^3^H]spiperone to the receptor could be detected. Four independent measurements were performed to obtain mean ± SEM values for K_D_ and B_max_. (**a**) construct 2: K_D_ = 0.31 ± 0.11 nM; B_max_ = 6.5 ± 2.2 receptors/cell. (**b**) construct 3: K_D_ = 0.14 ± 0.04 nM; B_max_ = 270 ± 12 receptors/cell.

The D_2_ receptor can be detected in two splice variants, the short D_2S_ and the long D_2L_ isoform, which have been suggested to be localized pre- and postsynaptically, respectively [26]. The only difference between the two forms is the lack of 29 amino acids within the third intracellular loop (ICL3) of D_2S_ [27]. In this study we used a modified bacterial expression vector pMAL-p5X (New England Biolabs, Frankfurt a. M., Germany), containing the lac instead of the tac promoter to express the gene of the D_2S_ isoform. In order to determine whether the receptor is expressed in *E. coli* in inclusion bodies or in a soluble form, several variants of the receptor were constructed (Figure 1) either without or with an N-terminal maltose binding protein (MBP) fusion (Constructs 1,2), and in addition with the C-terminal mCherry protein (Construct 3). 

All constructs harbored a C-terminal Strep-tag for purification. The periplasmatic MBP targets the N-terminus of the receptor to the inner membrane, thus the construct without MBP should lead to inclusion bodies. However, no D_2S_ protein could be detected for construct 1 in Western blots, using an anti-Strep-tag antibody (StrepMAB-Classic, IBA Lifesciences, Göttingen, Germany) indicating no expression or degradation of the receptor protein (data not shown). In contrast, using constructs 2 and 3, radioligand binding assays with whole bacterial cells confirmed expression of D_2S_ which is capable of ligand binding (Figure 1a,b). Furthermore, these results indicate that the radioligand is able to penetrate the outer membrane. In fact, the receptor population could be increased ~40-fold in the presence of the C-terminal fusion protein mCherry (Figure 1; Table 1). Analysis of the corresponding K_D_-values using the ligand [^3^H]spiperone by an unpaired T-test indicated that the obtained values are not statistically different (Table 1). 

**Figure 2:**
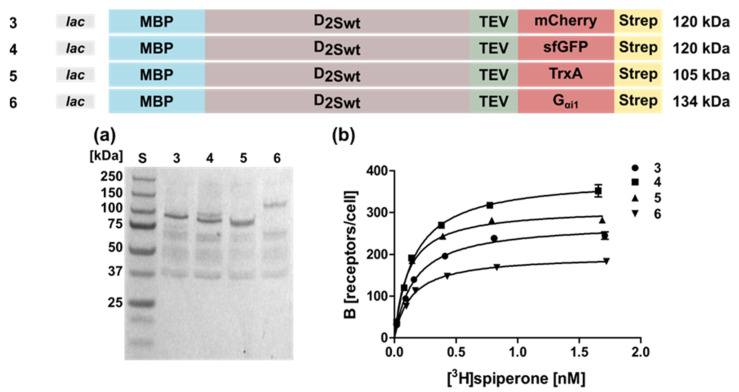
Expression and radioligand binding of D_2S_ wild type constructs with different C-terminal fusion proteins. The constructs expressed in *E. coli* strain TOP10 are shown on top along with the corresponding molecular weights. Expression was induced with 0.5 mM IPTG overnight. (**a**) Western blot against the N-terminal MBP. Aliquots were lysed and proteins were separated by SDS-PAGE using a 10% gel and transferred to a nitrocellulose membrane. Lane numbers correspond to the constructs shown above; S, molecular weight standard. The molecular weights of the standard proteins are shown on the left. (**b**) Binding of [^3^H]spiperone to *E. coli* whole cells expressing the D_2S_ wild type receptor. Numbers indicate the constructs shown on top.

In addition to mCherry, we fused the superfolder green fluorescent protein (sfGFP, Figure 2 Construct 4), thioredoxin A (Construct 5), or the G protein G_αi1_ (Construct 6) to the C-terminus. The receptor population per cell varied only slightly between 330 ± 21 for sfGFP and 190 ± 18 for G_αi1_ (Table 1). Similarly, the K_D_-values were comparable (Table 1). Taken together, the presence of a fusion protein appears to be indispensable, but not so much the type of the fusion protein.

Similar constructs using mCherry as the C-terminal fusion protein were made with the D_3_ receptor (Table 1), however, the expression did not reach the same high values as obtained for D_2S_ (see below).

### 2.2. Use of the Tac Promoter and Introduction of Thermostabilizing Mutations

**Figure 3:**
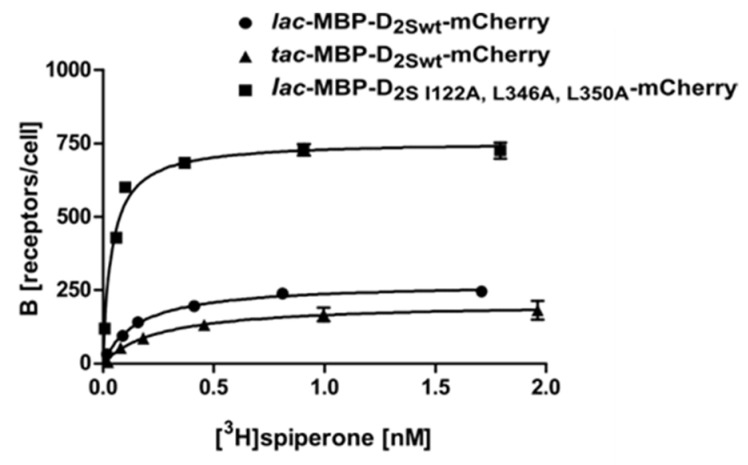
Radioligand binding assay with whole cells using D_2S_ expressed from different promoters and a thermostabilized variant. Binding of [^3^H]spiperone to the D_2S_ receptor: ● construct 3, ▲ construct 7, ■ construct 8. Exemplary, the binding curves of one measurement are shown. The K_D_- and B_max_-values determined are depicted in Table 1.

**Figure 4:**
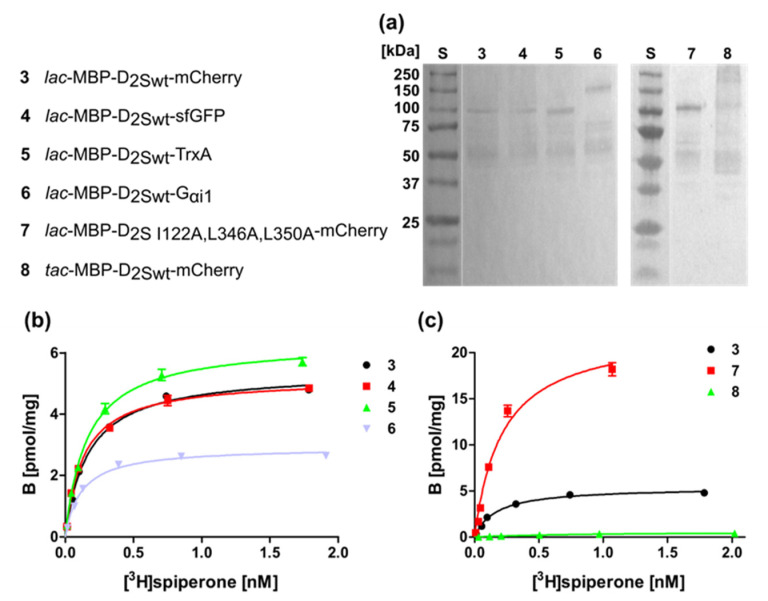
Membrane homogenates of different D_2S_ constructs. (**a**) Western blot against the N-terminal MBP. S, molecular weight standard, the molecular weights of the standard proteins are indicated on the left. Numbering of lanes corresponds to the numbers of the constructs shown on the left. (**b**,**c**) Binding of [^3^H]spiperone to the receptor in membrane homogenates. The numbering of the curves corresponds to the numbers of the constructs. K_D_- and B_max_-values are presented in Table 2.

For further investigations, we used Construct 3 (Figure 2) harboring the mCherry fusion at the C-terminus and analyzed if the stronger *tac* promoter improves expression in *E. coli* (Construct 8). However, overexpression from the tac promoter reduced the viability of the cells and thus resulted in less receptor molecules per cell (Figure 3; Table 1) and increased receptor degradation, as can be seen in the Western blot using membrane homogenates of different D_2S_ constructs (Figure 4a). 

The X-ray crystal structure of D_2L_ was solved recently using a mutated construct which included three thermostabilizing amino acid exchanges I122A, L375A, and L379A as well as the T4 lysozyme fused into ICL3 [28] (PDB: 6CM4). The modified gene was expressed in insect cells and purified for crystallization. It was shown before for the NTR1 receptor that thermostabilizing amino acid exchanges can also have an impact on the amount of receptor molecules in the *E. coli* membrane [23,29]. We thus wanted to analyze whether the corresponding amino acid exchanges in the D_2S_ receptor (I122A, L346A, L350A) also improve the production of correctly folded D_2S_ receptor in *E. coli*. As the T4 lysozyme in the ICL3 mainly supports crystallization but prevents an active conformation of the receptor, we avoided to engineer ICL3. 

Our results using the wild type construct 3 (lac-MBP-D_2Swt_-mCherry) and construct 7 (lac-MBP-D_2S-I122A,L346A,L350A_-mCherry) show that, besides thermostabilization, introduction of the amino acid exchanges I122A, L346A, and L350A resulted in a 3-fold increase of receptor molecules per cell. In combination with the C- terminal fusion protein, the exchanges have a strong impact on the amount of receptor leading to more than 730 receptor molecules per cell, as compared to only seven molecules per cell when using the wild type protein without a C-terminal fusion (Construct 2) (Figure 3; Table 1). This further implies that the *E. coli* system can be applied as a convenient primary screening system for mutants, as it is much cheaper and faster than eukaryotic systems. This procedure allows for the selection of mutants that are more effective in ligand binding. Only those will then be introduced into insect, yeast or mammalian cells for further analyses. It has been shown previously for the NTR1 receptor that improved variants selected in *E. coli* exhibit similar biochemical and biophysical properties in eukaryotic systems [30].

### 2.3. Isolation and Analysis of Membrane Homogenates after Expression of D_2S_

*E. coli* is a Gram-negative bacterium which possesses an inner and an outer membrane separated by the periplasmatic space. GPCRs can be incorporated into the inner membrane by the help of the signal peptide of the periplasmatic MBP that guides the N-terminus through the membrane. Although the dopamine receptor ligand spiperone can migrate through the outer membrane and bind to the dopamine receptor integrated in the inner membrane (Figure 1), some ligands might not be able to reach the periplasmatic space due to their size or charge. Thus, we set out to isolate membranes of bacterial cells expressing D_2S_ to test if they can also be used for ligand binding assays. 

We isolated the membranes of the bacterial strains harboring the D_2S_ constructs listed in Figure 4 and subjected the purified membranes to a Western blot directed against the N-terminal MBP protein to prove the presence of the D_2S_ variants (Figure 4a). In addition, the radioligand binding assay revealed ligand binding, which was comparable to whole cells, i.e., construct 7 containing the thermostabilizing amino acid exchanges exhibits the highest ligand binding capacity (Figure 4b,c; Table 2).

### 2.4. Expression of the D_3_ Receptor in the E. coli System Is Less Efficient

**Figure 5:**
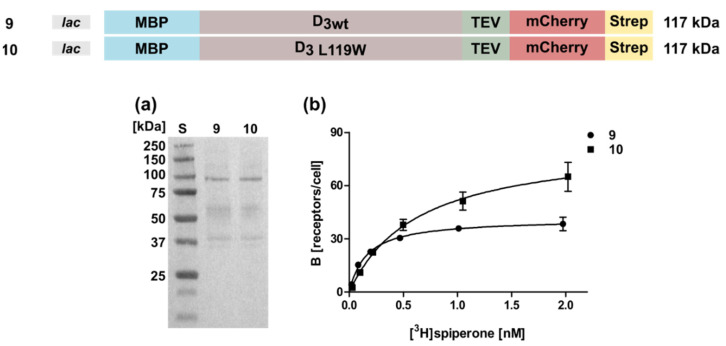
Expression and radioligand binding assays of D_3_ in whole cells. (**a**) Western blot against the N-terminal MBP. S, molecular weight standard, the molecular weights of the standard proteins are indicated on the left. Numbering of lanes corresponds to the numbers of the constructs shown on top. (**b**) Binding of [^3^H]spiperone to the receptor. The numbering of the curves corresponds to the numbers of the constructs. Construct 9: K_D_ = 0.52 ± 0.24 nM, B_max_ = 46 ± 3 receptors/cell; construct 10: K_D_ = 1.0 ± 0.14 nM, B_max_ = 69 ± 8 receptors/cell.

We then investigated whether the conditions optimized for the D_2S_ receptor could be used in a similar way for the expression of the D_3_ gene. However, expression of a D_3_ wild type construct harboring the MBP and mCherry at the N- and C-terminus, respectively, was less successful, leading to only 46 ± 3 receptor molecules per cell (Figure 5, Table 1). So far, for the D_3_ receptor only one thermostabilizing amino acid exchange L119W is known [31]. Compared to the corresponding wild type (Construct 9), introduction of L119W (Construct 10) increased the receptor population only slightly to 69 ± 8 receptors per cell (Figure 5, Table 1). However, the K_D_-value doubled from 0.52 ± 0.24 nM to 1.0 ± 0.14 indicating a negative impact of L119W on the [^3^H]spiperone binding affinity. Furthermore, similar to the results obtained for the D_2S_ receptor, exchanging the C-terminal fusion proteins had no significant effects (data not shown).

Taken together, the results for the D_3_ receptor demonstrate that the expression has to be optimized for every single GPCR individually and expression parameters cannot be transferred easily from one GPCR to another. Due to the negative effect of L119W on the K_D_-value, we proceeded our analyses with the wild type protein.

### 2.5. Optimization of the Induction Times

**Figure 6:**
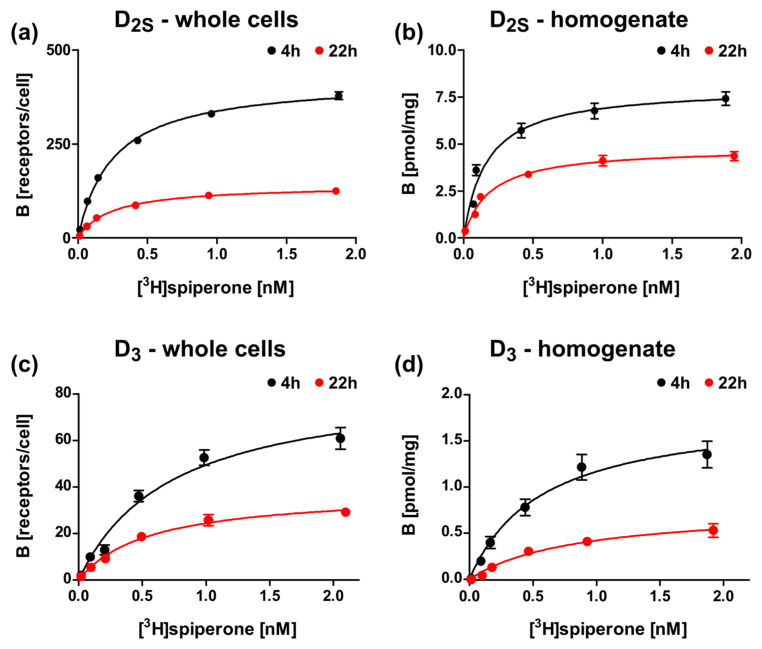
Radioligand binding assays with D_2S_ and D_3_ constructs after expression in *E. coli* with different induction times. Binding of [^3^H]spiperone to (**a**,**b**) lac-MBP-D_2Swt_-mCherry (Construct 3), or (**c**,**d**) lac-MBP-D_3wt_-mCherry (Construct 9) with (**a**,**c**) whole cells or (**b**,**d**) membrane homogenates after 4 h (black) or 22 h (red) induction with 0.5 mM ITPG.

To further optimize the expression, we tested different induction times for wild type D_2S_ and D_3_ constructs, lac-MBP-D_S2wt_-mCherry, and lac-MBP-D_3wt_-mCherry, respectively, and analyzed ligand binding for whole cells and for the membrane homogenates. As already shown above, the presence of receptors is significantly lower for the D_3_ construct, however the receptor population can be increased for both receptors by reducing the expression time from 22 h to 4 h (Figure 6, Table 3).

### 2.6. Establishment of a Method to Select Functional Thermostable Dopamine Receptor Variants in E. coli

We have shown above that we could significantly improve the production of the D_2S_ receptor in *E. coli* by introducing N- and C-terminal fusion proteins as well as thermostabilizing mutations. We also achieved higher B_max_ values for the thermostabilized L119W variant compared to the D_3_ wild type receptor (Figure 5), however the L119W exchange resulted in lower affinities for the ligand spiperone. Thus, search for thermostabilizing mutations which increase the receptor population in the membrane but do not impair ligand binding is required.

Directed evolution can be used to select for receptor variants that are more thermostable and bind a ligand with high affinity [30]. In brief, to achieve this goal, a receptor library is constructed using error-prone PCR. To select for improved receptor variants, the library is subjected to repeated FACS cycles by using a fluorescent ligand that binds to the receptor with high affinity. 

To test this approach, we used a fluorescent ligand NMP130, which was recently developed for the D_3_ receptor and used for analyses in human embryonic kidney (HEK) 293 cells [32]. Radioligand competition assays revealed an almost 20-fold higher affinity of the ligand NMP130 for the D_3_ receptor (0.76 nM) than for D_2S_ (15 nM) [32]. Thus, we tested the ligand with *E. coli* cells expressing D_3_.

**Figure 7:**
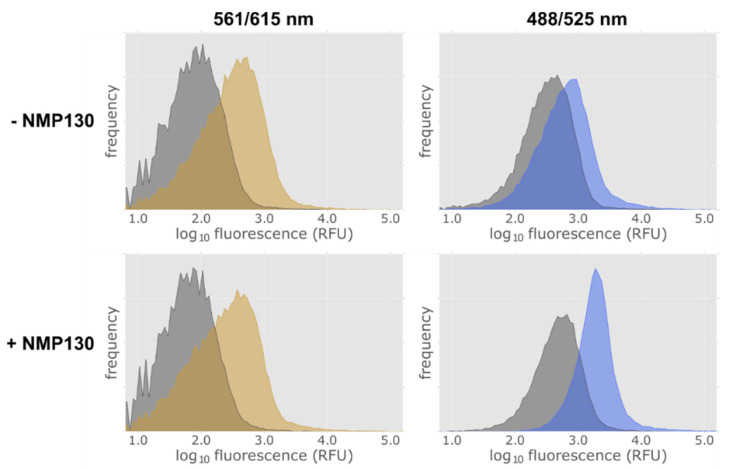
FACS analysis of D_3wt_ receptor expressing *E. coli* cells using the fluorescent ligand NMP130. Detection of construct 9 lac-MBP-D_3wt_-mCherry expression via the fusion protein mCherry at excitation and emission wavelengths of 561 and 615 nm in the absence (−) and presence (+) of the fluorescent ligand NMP130 (left panels). NMP130 was detected at excitation and emission wave lengths of 488 and 525 nm, respectively (right panels). Gray: without induction; yellow or blue, after induction with 0.5 mM IPTG for 4 h.

As NMP130 has only been tested in eukaryotic cells, it was necessary to determine if the ligand is able to cross the outer membrane of *E. coli* cells and bind to the D_3_ receptor. *E. coli* cells harboring lac-MBP-D_3wt_-mCherry (Construct 9) were induced for 4 h with 0.5 mM isopropyl-β-D-thiogalactoside (IPTG) and then subjected to FACS analysis. Uninduced cells were used as a control. Figure 7 proves that we can detect the expression of the receptor in *E. coli* via the fluorescence of the mCherry fusion protein using excitation and emission wave lengths of 561 nm and 615 nm, respectively. After induction, a fluorescence increase can be observed (Figure 7, left panels, yellow area). Binding of the ligand was detected at excitation and emission wave lengths of 488 nm and 525 nm. Without adding the ligand an increase of the fluorescence at 525 nm after induction could be detected (Figure 7, top right panel, blue area) which is due to an increased green cellular autofluorescence in *E. coli* cells upon induction with IPTG [33]. However, a distinct increase of the fluorescence at 525 nm after induction and addition of ligand could be observed (Figure 7, bottom right panel at the bottom, blue area).

To confirm that the binding of NMP130 to the D_3wt_ receptor was specific, we analyzed if the D_3_ ligand haloperidol [34] can displace NMP130. Using FACS, we could show that a 4000-fold excess of non-fluorescent haloperidol led to a strong reduction of the fluorescence at 525 nm indicating displacement of NMP130 (Figure S5).

**Figure 8:**
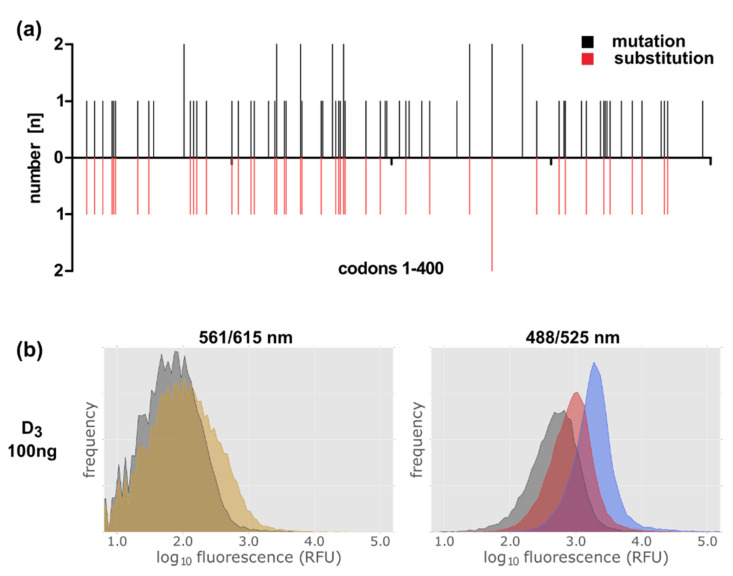
Construction of a D_3_ library by error-prone PCR. (**a**) Distribution of the mutations over the D_3_ gene. black: localization of point mutations; red: localization of mutations leading to amino acid substitutions. (**b**) FACS analysis of the gene library in the presence of the ligand NMP130. Detection of construct 9 lac-MBP-D_3_-mCherry variants’ expression via the fusion protein mCherry at excitation and emission wavelengths of 561 and 615 nm (left panel). NMP130 was detected at excitation and emission wave lengths of 488 and 525 nm, respectively (right panel). Gray: without induction; yellow after induction with 0.5 mM IPTG for 4 h; red: after induction + NMP130; blue: control, lac-MBP-D_3wt_-mCherry, D3 wild type construct after induction + NMP130.

After having proven with the D_3_ wild type protein that NMP130 is suitable for selecting new D_3_ variants by FACS analysis, we constructed a D_3_ receptor gene library via error-prone PCR. Oligodeoxynucleotides homologous to the regions adjacent to the D_3_ gene (Table S1, Oligos #1, #2) were used for the PCR reaction with the GeneMorph II Random Mutagenesis Kit. 1–5 mutations per gene were obtained using 23 PCR cycles and 100 ng template DNA. The library was cloned into the vector plasmid lac-MBP-mCherry via circular polymerase extension cloning (CPEC) (Table S1, Oligos # 1–4) [35,36]. We obtained ca. 7.3 × 10^5^ clones. To determine the number of mutations per gene and to verify that the mutations are distributed over the whole gene length we sequenced 22 independent clones (Figure 8a).

The library was then expressed in *E. coli* and, after addition of NMP130, subjected to FACS analysis (Figure 8). Full length receptor expression was controlled via the mCherry fusion at emission and excitation wavelengths of 561 and 615 nm, respectively (Figure 8b, left panel). In comparison to the expression of the wild type D_3_ receptor (Figure 7, left panels, yellow area), only a slight increase in fluorescence at 615 nm could be detected, because here, the introduction of various mutations can lead to incomplete non-functional receptor molecules, which might be degraded. Nevertheless, the increase in fluorescence at 525 nm indicates that receptor variants are present, which are still able to bind the ligand (Figure 8b, right panel, red peak). For comparison, an *E. coli* strain expressing the D_3_ wild type construct 9 was analyzed after NMP130 binding (Figure 8b, right panel, blue peak). Our result is a proof of principle showing that this method can be applied, e.g., for the selection of improved receptor variants that might be more thermostable as error-prone PCR and FACS selection can be repeated several times in order to enrich advantageous mutations [29,30].

## 3. Materials and Methods

### 3.1. Gene Cloning and Plasmid Preparation

All cloning experiments were carried out via Gibson assembly [37]. The genes coding for the human dopamine receptors D_2S_ (Uniprot: P14416) and D_3_ (Uniprot: P35462) (Eurofins Genomics, Ebersberg, Germany), adapted for *Trichoplusia ni* insect cells, were cloned into the modified vector pMAL-p5X (New England Biolabs, Frankfurt a. M., Germany), in which the *tac* promotor had been replaced by the weaker *lac* promotor. The constructs for the dopamine receptors were designed with and without N-terminal MBP (~43 kDa). In addition, all constructs harbored a tobacco etch virus (TEV) protease cleavage site and a Strep-tag at the C-terminus. In case of an additional C-terminal fusion protein, namely, mCherry (~28 kDa), sfGFP (~28 kDda), TrxA (~12 kDa), and Gα_i1_ (~40 kDa), the corresponding gene was fused between the coding region for the TEV protease cleavage site and the Strep-tag. Plasmids containing the genes for the fusion proteins mCherry and sfGFP were kindly provided by A. Möglich (University of Bayreuth, Bayreuth, Germany) and F. Bernhard (University of Frankfurt, Frankfurt, Germany), respectively. The vector pET-32a (Merck (Novagen), Darmstadt, Germany) was used as a template to amplify the gene for TrxA. The gene coding for the Gα_i1_ subunit (Uniprot: P63096) with the codon usage adapted for *Spodoptera frugiperda* was purchased from Eurofins Genomics (Ebersberg, Germany).

Mutations leading to the amino acid substitutions I122A, L346A and L350A for the D_2S_ receptor and L119W for the D_3_ receptor were introduced by site directed mutagenesis. 

### 3.2. Heterologous Gene Expression in E. coli

TOP10 (Invitrogen-Life Technologies, Darmstadt, Germany) harboring the corresponding plasmids for expression of the human dopamine receptors were incubated at 37 °C in 2x TY medium supplemented with 1% glucose and 100 mg/mL ampicillin until an OD600 of ~0.5 was reached. Gene expression was induced with 0.5 mM IPTG at 20 °C either for 4 h or 22 h. Cells were harvested by centrifugation at 4 °C and 8000× *g* for 20 min. The pellets were either processed immediately or stored at 20 °C until usage. 

### 3.3. Preparation of Membrane Homogenate

Cells were resuspended in lysis buffer (50 mM Tris/HCl, pH 7.4, 150 mM NaCl, 10% (*v/v*) glycerol) and incubated on ice after adding lysozyme and DNase I. After 20 min cells were lysed using a Microfluidizer (MFTI Corporation, Newton, MA, USA). Cell debris was removed by centrifugation at 4 °C and 6600× *g* for 30 min. The supernatant containing the membranes was subjected to ultracentrifugation at 4 °C and 120,000× *g* for 1 h. The membrane pellet was resuspended in 50 mM Tris/HCl, pH 7.4, 1 mM EDTA, 5 mM MgCl_2_ using a Dounce homogenizer. The total protein concentration of the membrane homogenate was determined with the Pierce™ BCA Protein Assay Kit (Thermo Fisher Scientific, Waltham, MA, USA).

### 3.4. Western Blot Analysis

Total protein of whole *E. coli* cells (2.5 × 10^7^) or membrane proteins (2 µg) of the membrane homogenates were separated by 10% Bis-Tris SDS-PAGE and transferred to a 0.45 µm nictrocellulose membrane (Sartorius, Göttingen, Germany). The membrane was blocked with 3% BSA in 1x TBS (20 mM Tris, pH 7.4, 150 mM NaCl), 0.1% (*v/v*) Tween20 for 1 h at room temperature. After washing the membrane (1x TBS, 0.1% (*v/v*) Tween 20) it was incubated with anti-MBP monoclonal antibody (1:10,000) (New England Biolabs, Frankfurt a. M., Germany), for 1 h at room temperature in 1x TBS, 0.2% (*w/v*) BSA, 0.1% (*v/v*) Tween 20. Anti-mouse IgE-alkaline phosphatase antibody (1:20,000) (Sigma-Aldrich, Taufkirchen, Germany) was added and the membrane was incubated further for 1 h at room temperature. The blot was developed using 4.2 mg/mL NBT and 2.1 mg/mL BCIP as a substrate in 100 mM Tris/HCl, pH 9.5, 100 mM NaCl, 5 mM MgCl_2_. 

### 3.5. Radioligand Binding Assay

Determination of the binding parameters of the radioligand [^3^H]spiperone to the various receptor constructs have been performed by saturation binding experiments based on the experimental conditions described previously [38,39]. In detail, *E. coli* cells were incubated at cell densities of 1 × 10^8^ cells/mL, 3 × 10^8^ cells/mL, and 1 × 10^9^ cells/mL together with [^3^H]spiperone (specific activity of 79 Ci/mmol, PerkinElmer, Rodgau, Germany) in linear concentrations in the range of 0.02 to 2.0 nM (final concentrations) in a volume of 200 µL in buffer (50 mM Tris, 5 mM MgCl_2_, 1 mM EDTA, 100 µg/mL bacitracin, and 5 µg/mL soybean trypsin inhibitor at pH 7.4) for 60 min at 37 °C. Binding reaction was terminated by filtration of the cell suspension on a GF/B filter mat followed by a drying step at 60 °C. Trapped radioactivity was determined by scintillation measurement using a microplate reader (MicroBeta Trilux, PerkinElmer, Rodgau, Germany). Membrane preparations were tested analogously by incubating 50 µg/mL of protein with the radioligand. Each experiment was performed in quadruplicates. Unspecific binding was determined in the presence of 10 µM of haloperidol. Analysis of receptor density (B_max_) and binding affinity of the radioligand (K_D_-value) for each construct was performed by applying the algorithms for one site binding (hyperbola) in Prism 6.0 (GraphPad, San Diego, CA, USA).

### 3.6. Fluorescent Activated Cell Sorting

The D_3_ receptor genes and the library were expressed in *E. coli* TOP10 for 4 h as described above. 2 × 10^8^ cells were centrifuged at room temperature and 7000× *g* for 4 min and the pellet was resuspended in 2.5 mL ice cold 1x TBS + protease inhibitor (P) (Roche, Basel, Switzerland). For the analysis of NMP130 binding, 2 × 10^8^ cells were centrifuged as before and the pellet was resuspended in 500 μL ice cold 1x TBS + P with 200 nM NMP130 and incubated on ice for ≥1.5 h. After centrifugation at 4 °C and 13,000 rpm for 90 s, the pellet was resuspended in 100 μL ice-cold 1x TBS + P. The suspension was transferred into a cytometric reaction vessel containing 2.4 mL 1x TBS + P and analyzed immediately by FACS.

For displacement experiments with haloperidol (Sigma-Aldrich, Taufkirchen, Germany, stock solution 10 mM in ethanol) 200 nM NMP130 and 800 µM haloperidol was added to the cells. In the control assays haloperidol was substituted by 8% (*v/v*) ethanol. All experiments were carried out in a S3e Cell Sorter (Bio-Rad, Watford, UK). The data were plotted with Python (Python version 3.8.2). The script was kindly provided by Prof. Dr. Andreas Möglich (University of Bayreuth).

### 3.7. Library Construction

The GeneMorph II Random Mutagenesis Kit (Agilent, Santa Clara, CA, USA) was utilized to amplify the gene for the D_3_ receptor via error-prone PCR using the oligonucleotides #1 and #2 (Supplementary Table S1). Twenty-three PCR cycles and 100 ng template DNA were used to obtain 1–5 mutations per gene. The template DNA was thereafter digested with the enzyme DpnI (20 U/50 μL) at 37 °C for 2 h. After purification via MinElute PCR Purification Kit (Qiagen, Hilden, Germany) the D_3_ gene library was further amplified with Phusion DNA polymerase (New England Biolabs, Frankfurt a. M., Germany) to obtain sufficient amounts of DNA for cloning.

The D_3_ gene library was cloned via circular polymerase extension cloning (CPEC) [35,36] into the modified vector pMAL-p5X containing a *lac* promotor and the fusion proteins MBP (N-terminus) and mCherry (C-terminus). The vector backbone fragment was amplified using touchdown PCR [40] and oligonucleotides #3 and #4 (Supplementary Table S1). 

80 µLof electrocompetent *E. coli* TOP10 cells were transformed with 10 µL of the CPEC reaction. Immediately, 910 µL SOC (Super Optimal broth with Catabolite repression) medium [41] was added and cells were shaken at 37 °C for 1 h. The cell suspension was transferred into 50 mL 2x TY medium, 3% glucose, 100 mg/mL ampicillin and shaken at 37 °C for 2 h. Next, the culture was filtered (5 µm, Sartorius, Göttingen, Germany) and the filtrate was centrifuged at 8000× *g* for 10 min. The cell pellet was resuspended with 2x TY medium, 3% glucose, 100 mg/mL ampicillin and shaken overnight at 30 °C. 2 × 10^9^ cells were pelleted (4 °C, 7000× *g*, 5 min), resuspended in 200 µL Hognees Freezing Medium [42], snap frozen in liquid nitrogen, and stored at 80 °C. 

## 4. Conclusions

For a better molecular understanding and characterization of the two human dopamine receptors D_2S_ and D_3_, heterologous expression systems can help to select for stabilized variants that can be used to investigate the proteins in their active forms. Here, we provide constructs for D_2S_ and D_3_ which allow for successful expression of the functional receptors in *E. coli*. This method can either be used to examine ligand binding properties of receptor variants or to screen for new ligands using ligand displacement. What is more, a directed evolution approach was established as a proof of principle for the selection of improved receptor variants. During this procedure, mutations will be introduced that can change the properties of the receptors, i.e., they can lead to higher expression levels, slower degradation and/or higher thermostability. The latter changes can result in changes in receptor dynamics. However, functional receptor variants can be generated and selected based on ligand binding properties. These variants can then be produced in sufficient quantities for structural studies. 

It has been shown previously that GPCR expression levels of ~13 pmol/mg are needed in order to obtain 1 mg of GPCR in 5 L culture [9]. Dodevski et al. estimated that around 3000–3500 GPCRs/cell correspond to approximately 1 mg/L functional receptor [16]. With the stabilized D_2S_ variant *lac*-MBP-D_2S-I122A,L346A,L350A_-mCherry we can already get around 740 receptors/cell or 13 ± 10 pmol/mg rendering it sufficient for receptor purification in a 4–5 L batch culture (Table 1 and Table 2). Using this variant in the directed evolution approach even higher yields of the receptor should be achieved. 

Although structures of the D_2S_ and D_3_ receptors are available, they can only provide information of a certain conformation either in the active or inactive form [28,31,43,44]. Thus, other methods like solution NMR should be carried out to get new insights into receptor dynamics. Expression of the receptors in *E. coli* facilitates isotope labeling of specific amino acid residues of the receptor which is absolutely required for NMR studies and cannot be done easily in eukaryotic cells [24]. Commercially available kits can be used for stereo-specific methyl group labelling or labelling of a specific amino acid. Depending on which and on how many amino acids are to be labeled the cost ranges from 300 to 1000 €/L culture. Alternatively, specifically labelled precursors or amino acids can be added to minimal medium, which reduces the cost to around 30 €/L for Ile/Leu/Val labelling to up to 400 €/L for Trp labelling. In either case, minimal medium has to be prepared with deuterated water and 2 g/L deuterated glucose which amounts to approximately 400 €/L and 30 €/L, respectively. In summary our work delivers the basis for the biophysical and conformational investigation of dopamine receptors in *E. coli*.

## Figures and Tables

**Table 1:** K_D_- and B_max_-values determined with radioligand binding assay with whole cells using different D_2S_ and D_3_ constructs after expression in *E. coli* for 22 h.

Construct Number	Construct Name	* K_D_ [nM]	* B_max_ [Receptors/Cell]
2	*lac*-MBP-D_2Swt_	0.31 ± 0.11	6.5 ± 2.2
3	*lac*-MBP-D_2Swt_-mCherry	0.14 ± 0.04	270 ± 12
4	*lac*-MBP-D_2Swt_-sfGFP	0.13 ± 0.04	330 ± 21
5	*lac*-MBP-D_2Swt_-TrxA	0.11 ± 0.03	280 ± 10
6	*lac*-MBP-D_2Swt_-G_αi1_	0.14 ± 0.01	190 ± 18
7	*lac*-MBP-D_2S- I122A,L346A,L350A_-mCherry	0.11 ± 0.04	740 ± 38
8	*tac*-MBP-D_2Swt_-mCherry	0.23 ± 0.03	190 ± 33
9	*lac*-MBP-D_3wt_-mCherry	0.52 ± 0.24	46 ± 3
10	*lac*-MBP-D_3 L119W_-mCherry	1.0 ± 0.1	69 ± 8

* K_D_-values in nM ± SEM, B_max_ values in receptors/cell ± SEM all derived from three to four repeats in quadruplicates.

**Table 2:** K_D_- and B_max_-values of *E. coli* membrane homogenates after expression of different D_2S_ constructs.

Construct Number	Construct Name	* K_D_ [nM]	* B_max_ [Pmol/mg]
3	*lac*-MBP-D_2Swt_-mCherry	0.11 ± 0.09	3.4 ± 2.9
4	*lac*-MBP-D_2Swt_-sfGFP	0.10 ± 0.04	3.3 ± 2.7
5	*lac*-MBP-D_2Swt_-TrxA	0.12 ± 0.06	4.1 ± 3.3
6	*lac*-MBP-D_2Swt_-G_αi1_	0.090 ± 0.05	2.0 ± 1.4
7	*lac*-MBP-D_2S-I122A,L346A,L350A_-mCherry	0.16 ± 0.09	13 ± 10
8	*tac*-MBP-D_2Swt_-mCherry	0.48 ± 0.39	0.23 ± 0.17

* K_D_-values in nM ± SD, B_max_.values in pmol/mg protein ± SD all derived from two repeats in quadruplicates.

**Table 3:** K_D_- and B_max_-values of *E. coli* whole cells and homogenates expressing the D_2S_ or D_3_ for 4 h and 22 h.

Construct Number	Construct Name	Whole Cells			
		^a^ K_D_ [nM]		^a^ B_max_ [receptors/cell]	
		4 h	22 h	4 h	22 h
3	*lac*-MBP-D_2Swt_-mCherry	0.34 ± 0.12 ^b^	0.14 ± 0.04 ^a^	410 ± 21 ^b^	270 ± 12 ^a^
9	*lac*-MBP-D_3wt_-mCherry	0.85 ± 0.14 ^b^	0.52 ± 0.24 ^a^	110 ± 42 ^b^	46 ± 3 ^a^
		**Homogenate**			
		**^b^ K_D_ [nM]**		**^b^ B_max_ [pmol/mg]**	
		4 h	22 h	4 h	22 h
3	*lac*-MBP-D_2Swt_-mCherry	0.20 ± 0.04 ^b^	0.11 ± 0.09 ^b^	8.1 ± 0.14 ^b^	3.4 ± 2.9 ^b^
9	*lac*-MBP-D_3wt_-mCherry	0.66 ± 0.13 ^b^	0.50 ± 0.21 ^b^	2.0 ± 0.21 ^b^	0.52 ± 0.25 ^b^

^a^ K_D_- values in nM ± SEM, B_max_-values in receptors/cell ± SEM all derived from three to four repeats in quadruplicates. ^b^ K_D_-values in nM ± SD, B_max_-values in pmol/mg protein ± SD all derived from two repeats in quadruplicates.

## Data Availability

The data are contained within this article.

## Supplementary Materials

The following are available online at https://www.mdpi.com/article/10.3390/ijms22168647/s1, Table S1: Oligodesoxynucleotides used for error prone PCR of D_3_ and CPEC; Figure S1: Expression analysis for D_2S_ expressed from different promoters and of a thermostabilized variant; Figure S2: Original image of the Western Blot presented in Figure 2; Figure S3: Original images of the Western Blots presented in Figure 4; Figure S4: Original image of the Western Blot presented in Figure 5; Figure S5: The D_3_ ligand haloperidol can displace the fluorescent ligand NMP130.
