# Supplementary material for: Optimizing the Expression of Human Dopamine Receptors in Escherichia coli
Source: Int J Mol Sci. 2021 Aug 11;22(16):8647. doi: 10.3390/ijms22168647 (PMC8395450; doi:10.3390/ijms22168647)
Supplement: Supplementary file 1 [file ijms-22-08647-s001.zip › ijms-1249774-supplementary.pdf]

Optimizing the Expression of Human Dopamine Receptors in *Escherichia coli*

Vanessa Boritzki[1], Harald Hübner[2], Anni Allikalt[2], Peter Gmeiner[2], Birgitta M. Wöhrl[1]

[1] Department of Biochemistry IV - Biopolymers, Universität Bayreuth, Universitätsstr. 30, D-95447 Bayreuth, Germany

[2] Department of Chemistry and Pharmacy, Medicinal Chemistry, Friedrich-Alexander-Universität Erlangen-Nürnberg, Nikolaus-Fiebiger Str. 10, 91058 Erlangen, Germany.

Supplementary Tables and Figures

Tables

Table S1. Oligodeoxynucleotides used for error prone PCR of D_3 and CPEC.

#	5´→3´
1	AAGACGCGCAGACTCATATG
2	CCTTGAAAATACAGGTTTTCAAGCTT
3	AAGCTTGAAAACCTGTATTTTCAAGGC
4	CATATGAGTCTGCGCGTCTTTCAG

Figures

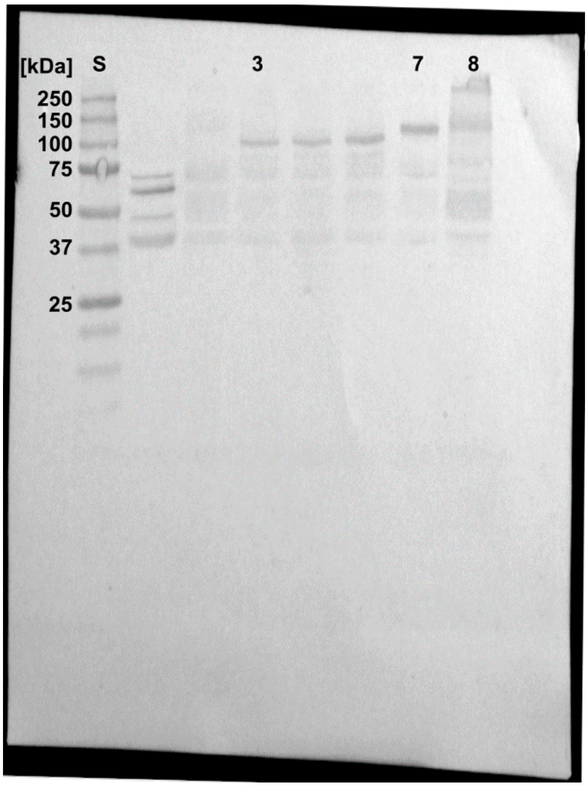

Figure S1. Expression analysis for D_{2S} expressed from different promoters and of a thermostabilized variant. Original image of the Western blot against the N-terminal MBP. S, molecular weight standard, the molecular weights of the standard proteins are indicated on the left. Numbers on top of the gel indicate construct numbers: **3**, *lac*-MBP-D_{2Swt}-mCherry; **7,** *tac*-MBP-D_{2Swt}-mCherry; **8,** *lac*-MBP-$D_{2S\ I122A,L346A,L350A}$-mCherry.

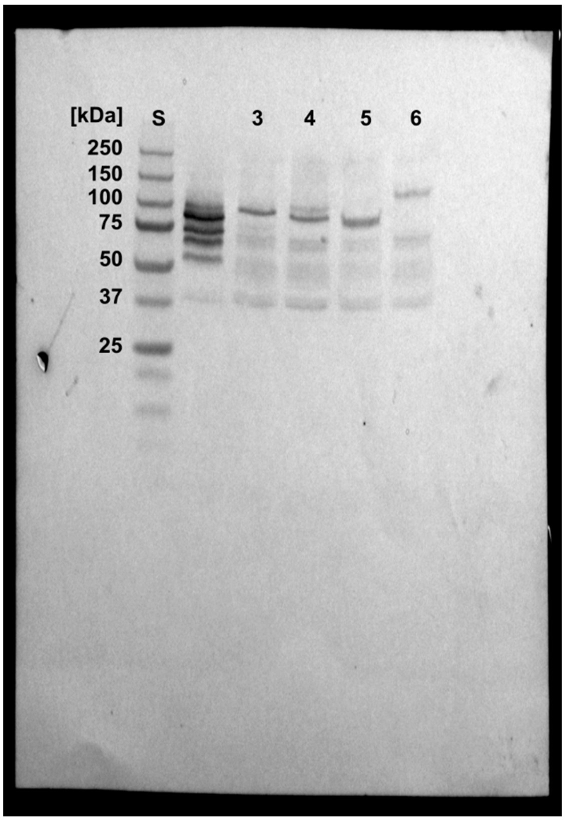

Figure S2. Original image of the Western Blot presented in Figure 2. Western Blot against the N-terminal MBP. S, molecular weight standard, the molecular weights of the standard proteins are indicated on the left. Numbers on top of the gel indicate construct numbers: **3** *lac*-MBP-D_{2Swt}-mCherry, **4** *lac*-MBP-D_{2Swt}-sfGFP, **5** *lac*-MBP-D_{2Swt}-TrxA, **6** *lac*-MBP-D_{2Swt}-$G_{\alpha i1}$.

(a)

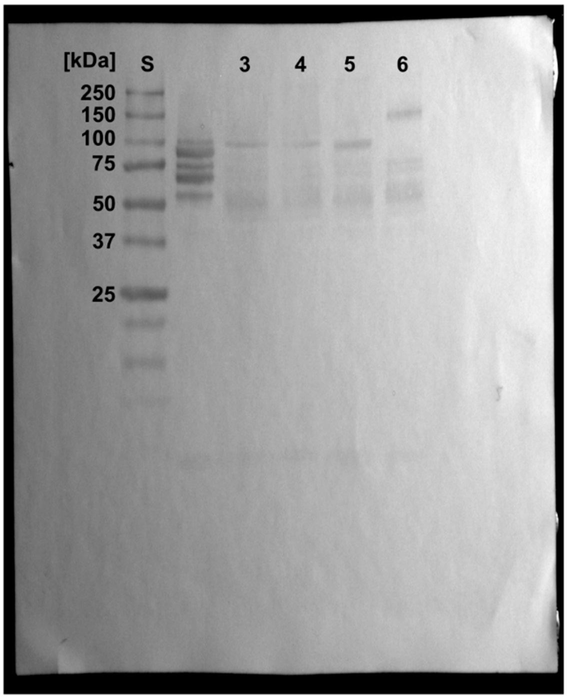

(b)

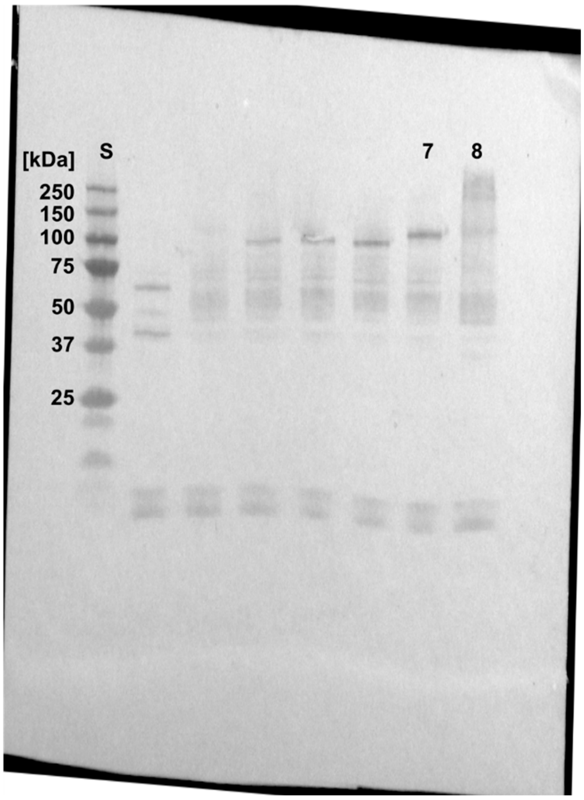

Figure S3. Original images of the Western Blots presented in Figure 4. Western Blots against the N-terminal MBP. S, molecular weight standard, the molecular weights of the standard proteins are indicated on the left. Numbers on top of the gel indicate construct numbers. **(a)** **3** *lac*-MBP-D_{2Swt}-mCherry, **4** *lac*-MBP-D_{2Swt}-sfGFP, **5** *lac*-MBP-D_{2Swt}-TrxA, **6** *lac*-MBP-D_{2Swt}-$G_{\alpha i1}$. **(b)** **7** *lac*-MBP-$D_{2S\ I122A,L346A,L350A}$-mCherry, **8** *tac*-MBP-D_{2Swt}-mCherry.

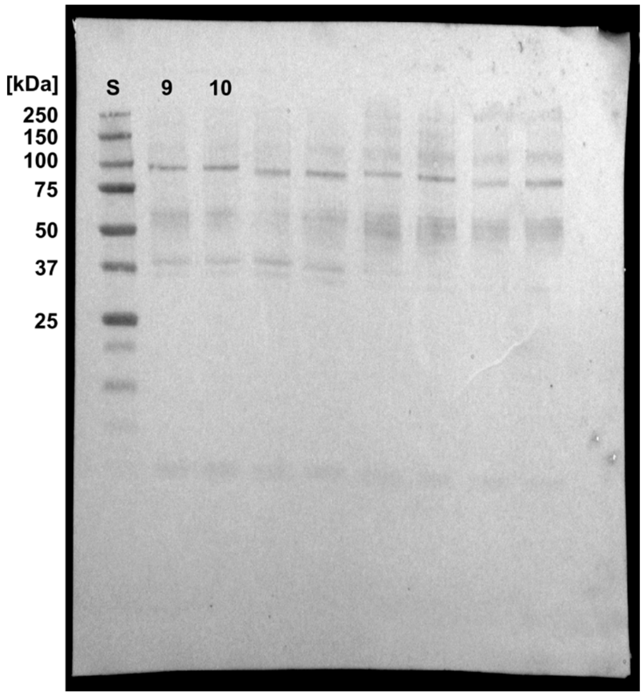

Figure S4. Original image of the Western Blot presented in Figure 5. Western Blot against the N-terminal MBP. S, molecular weight standard, the molecular weights of the standard proteins are indicated on the left. Numbers on top of the gel indicate construct numbers: **9** *lac*-MBP-D_{3wt}-mCherry, **10** *lac*-MBP- $D_{3\ L119W}$-mCherry.

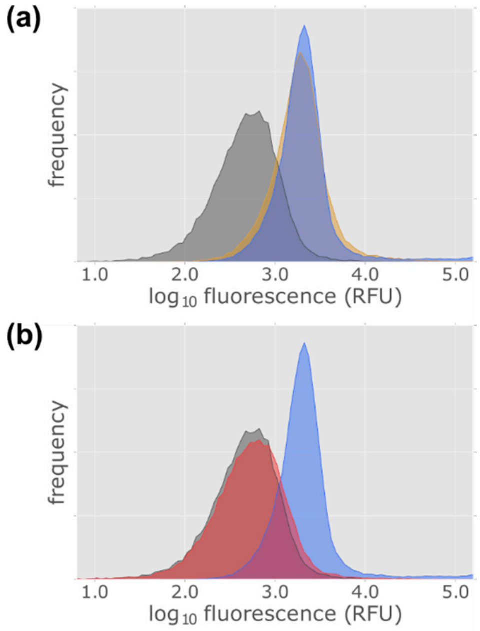

Figure S5. The D_3 ligand haloperidol can displace the fluorescent ligand NMP130. **(a)** FACS analysis at excitation and emission wave lengths of 488 and 525 nm showed that 8 % ethanol in the labeling reaction does not significantly alter binding of NMP130 to the D3 receptor (control). Grey: without induction, yellow: after induction with 0.5 mM IPTG for 4 h, blue: after induction with 0.5 mM IPTG for 4 h and labeling reaction containing 8 % ethanol (see Material & Methods for further details). **(b)** Specific binding of NMP130 to the D_{3wt} receptor was confirmed by displacement with haloperidol. Grey: without induction, blue: after induction with 0.5 mM IPTG for 4 h and labeling reaction containing 8 % ethanol (Fig. S5a), red: after induction with 0.5 mM IPTG for 4 h and labeling reaction containing a 4000-fold excess of haloperidol.
